# A painful tic convulsif due to double neurovascular impingement

**DOI:** 10.1007/s10194-011-0370-0

**Published:** 2011-08-04

**Authors:** G. Giglia, M. Romano, P. Paladino, V. Virzì, F. Narese, A. Palermo, B. Fierro, F. Brighina

**Affiliations:** 1 Department of Experimental Biomedicine and Clinical Neurosciences, University of Palermo, Via G. La Loggia, 1, 90129 Palermo, Italy; 2 Department of Neurology, Hospital “Villa Sofia”, Palermo, Italy; 3 Radiology Unit, Casa di Cura “Regina Pacis”, San Cataldo, CL Italy

**Keywords:** Painful tic convulsif, Trigeminal neuralgia, Hemifacial spasm, Double vascular impingement

## Abstract

Here we present the case of a 50-year-old man suffering from “painful tic convulsif”, on the left side of the face, i.e., left trigeminal neuralgia associated with ipsilateral hemifacial spasm. An angio-MRI scan showed a neurovascular confliction of left superior cerebellar artery with the ipsilateral V cranial nerve and of the left inferior cerebellar artery with the ipsilateral VII cranial nerve. Neurophysiological evaluation through esteroceptive blink reflex showed the involvement of left facial nerve. An initial carbamazepine treatment (800 mg/daily) was completely ineffective, so the patient was shifted to lamotrigine 50 b.i.d. that was able to reduce attacks from 4 to 6 times per day to 1 to 2 per week. Considering the good response to the drug, the neurosurgeon decided to delay surgical treatment.

## Introduction

“Painful tic convulsif” is a rare condition characterized by paroxysmal irritative dysfunction of the V and VII ipsilateral cranial nerves [1]. Since the first description in 1920 by Cushing [2] only few cases were reported in literature. Most of them were caused by masses or by a singular vascular loop in the posterior fossa, involving both the facial and trigeminal nerves [3]. Even if this condition is considered rare, we think that a considerable number of cases might escape notice unless careful history, neuroimaging and neurophysiological studies are performed.

## Case report

We report on a 50-year-old Italian man. His chief complaints were episodes of pain in infraorbital region and muscular twitches of ipsilateral hemiface. He had no family history for headache or similar pain and there was nothing remarkable in his past medical history.

## Current medical history

The patient presented to our headache center with a 2-year history of pain in the left infraorbital region and subsequent ipsilateral facial spasm. At onset patient experienced short episodes of excruciating pain in the left infraorbital region. Three months later an ipsilateral facial spasm took place. Patient referred to his general practitioner and underwent a brain MRI scan, that was normal, then a diagnosis of “trigeminal neuralgia” was made and the patient started a trial with carbamazepine (CBZ) (800 mg/daily) that was ineffective. Pain episodes were described as “always the same, very intense, sometimes unbearable, localized exactly under my left eye”, lasting about 30 s and presenting four to six times per day. No trigger areas were reported. The facial twitch started about 3 months after the beginning of pain and was described as “involuntary grimace unrelated to pain”.

## Current illness

Physical examination was normal; blood pressure: 120/70 mmHg and heart rate: 80 bpm. Also routine laboratory data were normal. Neurological examination revealed a mild peripheral palsy of the VII nerve with very frequent hemifacial spasms in the left side. No other neurological abnormalities were present.

## Neuroimaging

### Angio-MRI scan

**Fig. 1:**
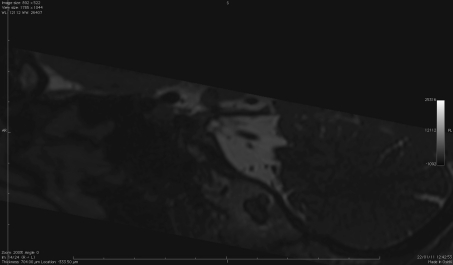
Parasagittal scan for the study of the Vth nerve

**Fig. 2:**
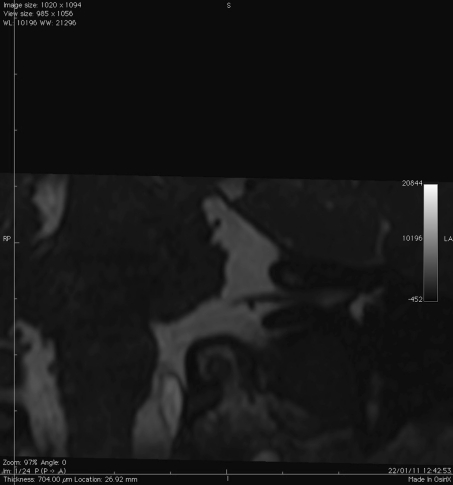
Coronal scan for the study of the VIIth nerve

A 1.5-T MRI scan was performed with FSE sequences, SE and FLAIR, T1 and T2-weighted. TOF3D sequences were also collected to study the vascular tree. No abnormalities of brain parenchyma, parenchymal cerebellar or brainstem were found. The MR angiographic examination and the thin layer study of the cranial base showed: close contiguity of the trigeminal nerve with left medial ipsilateral posterior cerebellar artery and inferiorly with a small venous vessel (Fig. 1); close contiguity of the left VII and VIII cranial nerve with ipsilateral posterior inferior cerebellar artery (Fig. 2).

### Neurophysiology

A blink reflex examination, performed stimulating the supraorbital branch of trigeminal nerve and recording from orbicular eye muscles, showed delayed ipsilateral R1 and R2 responses with normal contralateral R2 stimulating the left side whereas the inverse pattern, with normal ipsilateral R1 and R2 responses with delayed contralateral R2 was found after right stimulation; thus, indicating an incomplete left facial nerve lesion. A laser evoked potential (LEP) examination showed normal responses from both left and right infraorbital region.

### Diagnosis

A diagnosis of “painful tic convulsif due to double vascular impingement” was made as hemifacial spasms occurred in a patient fully satisfying IHS criteria [4] for trigeminal neuralgia.

### Therapy

As the initial CBZ treatment (800 mg/die) was unsuccessful, we started lamotrigine (LTG) with slow titration (adding 25 mg per week) to the final dose of 50 mg b.i.d. LTG led to a satisfactory control of pain with a reduction of episodes from 4–6 per day to 1–2 per week. Considering the good response to drug the neurosurgeon decided to delay surgical microvascular decompression.

## Discussion

“Painful tic convulsif” is a rare disorder first described by Cushing [2] in three patients, characterized by a double ipsilateral trigeminal and facial irritative syndrome. The commonest cause is vascular compression by ectatic vertebrobasilar artery, even if it may also arise from neoplasms [3, 5]. Pathophysiology of cranial nerve compression rizopathies is still debated as peripheral and central theories are equally considered. Demyelization of axons and subsequent ephactic transmission, along with hyperactivity of neurons in the brainstem nuclei, are probably able to induce spontaneous activation of nerves with sensory (V) and motor (VII) symptoms [3]. In our patient a mild deficit of VII cranial nerve was also clinically and electrophysiologically evident, suggesting that initial damage of neural fibers could also be present. Conversely the trigeminal nerve showed only functional impairment. Most “painful tic convulsif” cases reported in literature presented with involvement of VII preceding the V [5], while in our patient the inverse pattern occurred. This sequence could lead us to consider the facial spasm as due to pain, resembling a simple trigeminal neuralgia, previously called “tic doloreux” by French authors. However, the impairment of the VII cranial nerve, as showed by clinical and neurophysiological findings is not in favour of such interpretation and should be regarded as an independent phenomenon likely due to the neuro-vascular impingement. A meticulous history recording and prolonged observation during physical exam should be performed in all cases of trigeminal neuralgia to avoid underestimation of a possible “painful tic convulsif”.
